# Evaluation of healthcare workers’ compliance with contact isolation precautions and associated factors in pediatric and pediatric surgery intensive care units

**DOI:** 10.1017/ash.2026.10902

**Published:** 2026-10-15

**Authors:** Gülsüm İclal Bayhan, Leeyan Alghoosen, Yazan Darwish, Lara Maraqa, Esra Koçkuzu, Vildan Selin Çayhan, Ümran Erden, Nurdan Kaykı Aksoy, Serhat Emeksiz

**Affiliations:** 1 Faulty of Medicine, Department of Pediatric Infectious Diseases, Ankara Yildirim Beyazit University, Ankara, Türkiye; 2 Faulty of Medicine, Ankara Yıldırım Beyazit University, Ankara, Türkiye; 3 Department of Pediatric Intensive Care Unit, Ankara Bilkent City Hospital, Children’s Hospital, Ankara, Türkiye; 4 Department of Pediatric Surgery, Ankara Bilkent City Hospital, Children’s Hospital, Ankara, Türkiye; 5 Department of Infection Control Committee, Ankara Bilkent City Hospital, Children’s Hospital, Ankara, Türkiye; 6 Department of Pediatric infectious Diseases, Trabzonsity, Faculty of Medicine, Karadeniz Technical University, Trabzon, Türkiye

## Abstract

**Objective:**

To evaluate healthcare workers’ (HCWs) adherence to contact precautions (CP) in pediatric intensive care settings and to identify factors associated with compliance.

**Design:**

Prospective observational study.

**Setting:**

Pediatric intensive care units (PICUs) and a pediatric surgical intensive care unit (PSCU) at a tertiary care hospital.

**Participants:**

Healthcare workers involved in patient care activities in PICUs and PSCU.

**Methods:**

HCWs were directly observed during patient care procedures. Adherence to CP measures was evaluated using a five-step checklist, and compliance levels were categorized as low, moderate, or high. Associations between compliance and potential influencing factors were analyzed.

**Results:**

A total of 367 observations were performed. High compliance was observed in 30.4% of procedures, moderate compliance in 54.2%, and low compliance in 15.4%. Compliance levels were significantly associated with professional category and ICU unit, whereas no significant association was found with the type of patient care activity or microorganism type.

**Conclusions:**

Compliance with CP was suboptimal and varied according to professional category and ICU unit. These findings suggest that adherence is influenced by both individual and unit-level factors. Targeted, context-specific interventions and continuous education may improve adherence to infection control measures.

## Introduction

Contact precautions (CP) are widely implemented infection prevention and control (IPC) measures aimed at reducing the transmission of infectious agents that spread via direct or indirect contact. Since their formal introduction in 1996, CP have been recommended for patients infected or colonized with pathogens of particular clinical or epidemiological importance.^1,2^ Pathogens requiring CP commonly include multidrug-resistant Gram-positive and Gram-negative organisms, *Clostridioides difficile*, and several viral agents such as respiratory syncytial virus, human metapneumovirus, adenovirus, rotavirus, norovirus, and parainfluenza virus.^3^ The increasing number of pathogens considered “epidemiologically important,” together with variability in local epidemiology, may complicate the consistent and sustainable implementation of CP in clinical practice.^4^

Despite their long-standing use, CP remain insufficiently standardized across healthcare settings. Significant variability exists not only between institutions but also within the same institution, reflecting differences in local epidemiology, MDRO types, and clinical practices.^5–7^ This heterogeneity extends to decisions regarding the initiation, implementation, and discontinuation of CP, highlighting a lack of consensus in IPC strategies at both national and international levels.^7^ Morever, evidence regarding their effectiveness and implementation in pediatric populations remains limited, with most available data derived from adult healthcare settings.^8–11^

Adherence to CP by healthcare workers (HCWs) can be challenging; increased workload, particularly in settings with a high burden of isolated patients, has been associated with lower adherence to CP.^12^ Additionally, the application of CP is not without consequences. Previous studies have demonstrated that isolation practices may lead to reduced HCW–patient interactions, increased adverse events, and negative psychological effects on patients, including feelings of stigma and decreased self-esteem.^2^ These measures are also associated with increased healthcare costs, greater consumption of personal protective equipment (PPE), and organizational constraints.^13^

In recent years, growing evidence has questioned the routine use of CP for MRSA and VRE. Discontinuation of CP for MRSA and VRE has been associated with a reduction in non-infectious adverse events, without an increase in MRSA/VRE infection rates.^8,11,14^ Similar findings have been observed for extended-spectrum beta-lactamase (ESBL)-producing organisms, where cessation of CP did not significantly impact transmission dynamics.^15,16^ These findings have prompted some institutions to revise their policies, shifting toward enhanced standard precautions, particularly improved hand hygiene, rather than routine use of gowns and gloves.^14^

Effective implementation of CP depends on consistent HCW adherence and supportive institutional systems that facilitate appropriate infection prevention practices. Importantly, CPs should not be evaluated in isolation, as their effectiveness and sustainability depend largely on how they are integrated with other infection prevention interventions. In recent years, increasing attention has been given to multimodal and context-specific approaches that combine CP with complementary strategies, including hand hygiene, environmental cleaning, HCW education, antimicrobial stewardship, and audit and feedback.^17–19^ These approaches emphasize the importance of tailoring infection prevention strategies to local epidemiology, patient populations, and institutional resources rather than relying uniformly on CP alone.

Despite these considerations, adherence to CP and the factors influencing compliance among HCWs in pediatric healthcare settings remain insufficiently characterized.^8–11^ Understanding barriers to appropriate CP use is essential for identifying specific gaps in current practice and developing effective, targeted improvement strategies. Therefore, this study aimed to evaluate HCWs’ adherence to CP and identify factors associated with non-adherence in pediatric and pediatric surgery intensive care units. By identifying modifiable barriers and areas of suboptimal practice, this study seeks to provide baseline evidence to support institutional quality improvement initiatives, inform the development of context-specific infection prevention policies and standards, and guide targeted interventions to optimize CP practices.

## Materials and methods

This prospective observational study was conducted to evaluate HCWs’ adherence to CP measures among patients under contact isolation in pediatric intensive care units (PICUs) and the pediatric surgical intensive care unit (PSCU). Ethical approval was obtained from the ethics committee of Ankara Bilkent City Hospital (approval number: TABED 2–26–1927).

### Settings

The study took place in the Children’s Hospital of Ankara Bilkent City Hospital and included observations in three intensive care units: two PICUs and one PSCU, each with a capacity of 16 beds. All patient rooms were single-occupancy rooms with glass partitions.

PICU 1 and PICU 2 shared the same consultant physician team and had the same chief physician. Resident physicians rotated monthly between units, resulting in changes to the resident staff each month. In contrast, nurses and other healthcare staff assigned to each PICU remained constant throughout the study period. PICU 1 and PICU 2 admitted children aged 1 month to 18 years, with similar patient populations and the same bed capacity. Both units managed critically ill children with a broad range of diagnoses, including sepsis, multiorgan dysfunction, trauma, postoperative neurosurgical and orthopedic conditions, and respiratory tract infections. Children with unstable vital signs requiring intensive monitoring, invasive or non-invasive mechanical ventilation, and/or inotropic support were admitted to these PICUs.

The PSCU provides specialized intensive care for surgical patients aged 0–18 years who require close postoperative monitoring or advanced organ support due to major surgery, acute surgical conditions, trauma, or postoperative complications. The unit manages children with congenital and acquired surgical disorders, including those undergoing complex thoracic, abdominal, colorectal, urological, and oncological procedures. Depending on clinical severity, patients receive Level II or Level III intensive care support, ranging from continuous cardiorespiratory monitoring and non-invasive respiratory support to invasive mechanical ventilation, advanced hemodynamic monitoring, vasoactive therapy, and multidisciplinary management of critically ill surgical patients.

### Contact isolation precautions

CP were implemented according to CDC recommendations for patients colonized or infected with epidemiologically important pathogens, including multidrug-resistant organisms (MDROs), *Clostridioides difficile*, and selected viral pathogens.^3^ Targeted MDROs included methicillin-resistant *Staphylococcus aureus* (MRSA), vancomycin-resistant *Enterococcus* (VRE), carbapenem-resistant *Enterobacterales* (CRE), multidrug-resistant and carbapenem-resistant *Pseudomonas aeruginosa*, multidrug-resistant and carbapenem-resistant *Acinetobacter baumannii*, and *Candidozyma auris*. CP were not applied for ESBL-producing *Enterobacterales*. Active surveillance to identify asymptomatic colonization was not routinely performed. Isolation decisions were based on clinical culture results. Both colonized and infected patients were managed under CP.

Patients requiring CP were identified through a daily review of microbiology culture results by the infection control nurse assigned to the relevant intensive care unit. When a pathogen requiring contact precautions was identified, the infection control nurse activated a contact precaution alert for the patient in the hospital’s electronic health record (EHR) system. This alert was visible to physicians and nurses in the relevant intensive care unit whenever they accessed the patient’s electronic medical record. In addition, the infection control nurse notified the responsible nurse by telephone. Appropriate isolation signage was also placed at the entrance of the patient’s room.

### Observation and data collection

Observations were performed by three physicians who received prior training on CP procedures during the study planning phase. To improve consistency between observers, all observers received standardized training before the study, and discrepant observations were discussed collectively until consensus was reached. Although observations were not concealed, they were conducted during routine care to minimize behavioral modification.

HCWs entering the rooms of patients under CP and preparing for patient contact were directly observed. Adherence to CP measures was evaluated using a five-step checklist: 1) hand hygiene before patient contact (hand washing or use of alcohol-based hand rub); 2) use of a non-sterile, long-sleeved gown; 3) use of disposable gloves; 4) appropriate removal of personal protective equipment (gloves removed first, followed by gown, with proper disposal); 5) hand hygiene after patient contact. Each step was scored as 1 (compliant) or 0 (non-compliant), yielding a total score ranging from 0 to 5. Compliance levels were categorized as low (0–2), moderate (3–4), and full (5).

Observations were conducted at randomly selected time periods during routine clinical care, including different shifts and days. The following variables were recorded: HCW professional category (nurse, physician, intern, cleaning staff, or other personnel, including radiology technicians and radiologists), sex, ICU, unit type, time of observation (before or after 12:30 PM), and type of patient care activity. Radiology technicians and radiologists were not permanent members of the ICU staff; they visited the units to perform bedside radiographic and ultrasonographic examinations. Patient care activities were categorized into four groups: 1) minimal physical contact; 2) non-invasive physical contact; 3) invasive procedures or contact with body fluids, and 4) aerosol-generating procedures. Detailed classifications are provided in Supplementary Table 1.

Observations were not restricted to patients with microbiologically confirmed infections. Patients without an identified causative pathogen were also included when CP had been implemented based on clinical indications. These indications included purulent or draining wounds, diarrhea without an identified causative organism, and clinical sepsis without a microbiologically confirmed etiology. Among these patients, CP were initiated empirically based on the clinical presentation and institutional infection prevention protocols, irrespective of subsequent microbiological confirmation.

### Statistical analyses

The sample size was calculated based on a previously reported compliance rate of 39.5%. Assuming a 95% confidence level and a margin of error of 5%, the minimum required number of observations was determined to be 367.^20^

Statistical analyses were performed using SPSS version 26.0. Descriptive statistics were presented as frequencies and percentages. Associations between categorical variables and compliance levels were evaluated using the χ^2^ test. Only variables with *P* < .05 in univariate analysis were included in the ordinal logistic regression model to assess their independent effects on compliance levels. Results of the regression analysis were expressed as odds ratios (ORs) with 95% confidence intervals (CIs). Model fit was assessed using Nagelkerke’s R^2^. A *P* value of <.05 was considered statistically significant.

For the additional analysis of pathogen-specific compliance, the original compliance score was used as an ordinal outcome to avoid loss of statistical power resulting from further categorization into low, medium, and full compliance groups. Compliance scores across pathogen categories were compared using the Kruskal–Wallis test.

## Results

A total of 367 observations were performed. High compliance was observed in 112 (30.4%) procedures, moderate compliance in 200 (54.2%), and low compliance in 57 (15.4%). Factors potentially associated with compliance levels are presented in Table 1. Univariable analysis showed statistically significant associations between contact isolation compliance levels and professional category (*P* = .022), ICU unit (*P* < .001), and ICU type (*P* = .014). No significant associations were found for sex, time of observation, microorganism type, or type of patient contact activity (Table 1).

**Table 1.** Contact precaution compliance levels among healthcare workers according to demographic, professional, and clinical characteristics Table 1 long description.

		Low compliance (n:57)	Medium compliance (n:200)	Full compliance (n:112)	
Sex n (%)	Female	38 (14.7)	150 (57.9)	71 (27.4)	.081
	Male	19 (17.3)	50 (45.5)	41(37.3)	
Professional category n (%)	Nurse	30 (11.8)	141 (55.3)	84 (32.9)	0.022
	Cleaner	8 (21.1)	20 (52.6)	10 (26.3)	
	Doctor	12 (24)	28 (56)	10 (20)	
	Intern doctor	4 (22.2)	6 (33.3)	8 (44.4)	
	Other personnel	3 (37.5)	5 (62.5)	0 (0)	
Contact Time n (%)	12:30 and earlier	23 (11.6)	110 (55.3)	66 (33.2)	.066
	12:31 and later	34 (20)	90 (52.9)	46 (27.1)	
Microorganisms n (%)	Gram-negative	13 (8.8)	88 (59.9)	46 (31.3)	.112
	Gram-positive	17 (21.0)	37 (45.7)	26 (33.3)	
	Candidozyma auris	0 (0)	4 (57.1)	3 (42.9)	
	Viruses	19 (18.4)	54 (52.4)	30 (29.1)	
ICU n (%)	PICU 1	37 (25.9)	63 (44.1)	43 (30.1)	*P* < 0,001
	PICU 2	8 (4.3)	121 (64.4)	59 (31.4)	
	PSCU	12 (31.6)	16 (42.1)	10 (26.3)	
ICU Type n (%)	Pediatric	45 (13.6)	184 (55.6)	102 (30.8)	.014
	Surgical	12 (31.6)	16 (42.1)	10 (26.3)	
Types of patient contact activities	Minimal physical contact	10 (22)	27 (60)	8 (17.8)	.139
	Non-invasive physical contact	22 (19.1)	58 (50.4)	35 (30.4)	
	Invasive procedures or contact with body fluids	15 (10.1)	86 (58.1)	47 (31.8)	
	Aerosol-generating procedures	10 (16.4)	29 (47.5)	22 (36.1)	

ICU, Intensive cure unit; PICU 1, Pediatric intensive care unit 1; PICU 2, Pediatric intensive care unit 2; PSCU, Pediatric surgical care unit.

An ordinal logistic regression analysis was performed to evaluate the independent effects of professional category and ICU unit on compliance levels. The model explained a modest proportion of the variance in compliance levels (Nagelkerke *R* ^2^ = .072). Using “other personnel” as the reference category, nurses were significantly more likely to have higher compliance levels (OR: 4.78; 95% CI: 1.20–19.08; *P* = .027). Similarly, intern doctors had a significantly higher likelihood of being in a higher compliance category (OR: 5.81; 95% CI: 1.14–29.60; *P* = .034). No statistically significant associations were observed for physicians or cleaners. Regarding ICU units, and using PSCU as the reference category, HCWs in PICU 2 had a significantly higher likelihood of higher compliance (OR: 2.73; 95% CI: 1.37–5.43; *P* = .004). No significant difference was observed between PICU 1 and PSCU (*P* = .212) (Table 2).

**Table 2.** Ordinal logistic regression analysis of factors associated with contact isolation compliance levels Table 2 long description.

Variables	β	OR(95% CI)	p value
Nurse vs other personnel	1.565	4.78 (1.20–19.08)	.027
Cleaner vs other personnel	1.126	3.08 (.70–13.61)	.137
Doctor vs other personnel	.806	2.24 (.52–9.61)	.278
Intern vs other personnel	1.760	5.81 (1.14–29.60)	.034
PICU 1 vs PSCU	0,451	1.57 (.77–3.19)	.212
PICU 2 vs PSCU	1.003	2.73 (1.37–5.43)	.004

An ordinal logistic regression model was applied in the table. The reference categories were “other” for personnel category other” and “PSCU” for ICU.

Pathogen-specific information was unavailable for 31 patients. These patients remained part of the overall study population but were excluded from pathogen-specific subgroup analyses. Pathogen-specific compliance scores are summarized in Table 3. No statistically significant difference was observed in median compliance scores across causative pathogen groups (*P* = .057).

**Table 3.** Distribution of compliance scores according to the identified pathogens Table 3 long description.

Identified pathogen	n	Median (range)
Pseudomonas spp. (MDR/CR)	133	4 (3–5)
VRE	32	3.5 (2–5)
Respiratory viruses	103	4 (3–5)
CRE	13	4 (3–5)
MRSA	29	4 (2–5)
Scarlet fever	20	4 (4–5)
Acinetobacter spp. (MDR/CR)	1	1 (1–1)
Candidozyma auris	7	4 (3–5)

Kruskal–Wallis test, χ^2^ = 13.676, df = 7, *P* = .057.

CRE, carbapenem-resistant Enterobacterales; MDR/CRE, multidrug-resistant or carbapenem-resistant; MRSA, methicillin-resistant Staphylococcus aureus; VRE, vancomycin-resistant Enterococcus.

## Discussion

In this prospective observational study, HCWs’ adherence to CP measures was evaluated in pediatric intensive care settings, and factors associated with compliance levels were identified. Overall compliance was suboptimal, with only one-third of observations demonstrating full adherence, while the majority of encounters were classified as moderate compliance. Nurses and intern doctors were more likely to demonstrate higher compliance compared with other occupational groups. In addition, despite comparable structural and organizational characteristics between PICU 1 and PICU 2, HCWs in PICU 2 demonstrated significantly higher adherence to CP measures, suggesting that unit-specific behavioral or cultural factors may influence compliance. Furthermore, adherence was not associated with the type of patient care activity or microorganism type, indicating that CP practices were applied more as routine behaviors related to room entry rather than according to procedural risk or transmission characteristics.

In this study, adherence to CP was highest among nurses. This finding is consistent with previous studies suggesting that nurses demonstrate better compliance with infection control measures than physicians.^21–23^ HCWs with more frequent patient contact and structured training in infection control tend to exhibit higher adherence. Nurses, in particular, are continuously engaged in bedside care and are often more familiar with institutional protocols, which may explain their higher compliance rates. The relatively high compliance observed among interns may be attributed to their recent training and closer supervision during clinical practice. When occupational groups were compared, cleaning staff had the highest workload. Staff shortages, heavy workload, and time pressure are well-known barriers to adherence.^24–25^ In this context, the lower compliance observed among cleaning staff may be explained by their higher workload. These findings highlight the need for targeted interventions, particularly ensuring adequate staffing levels to improve adherence among high-workload groups.

In the present study, however, PICU 1 and PICU 2 had comparable workload, staff-to-patient ratios, and patient characteristics and shared the same consultant physician team and department head. Despite these similarities, HCWs in PICU 2 demonstrated significantly higher compliance than those in the PSCU, whereas no significant difference was observed between PICU 1 and PSCU. HCWs working in PICU 2 were approximately 2.7 times more likely to be in the high-compliance group compared with those working in the PSCU, while no significant difference was found between PICU 1 and PSCU. Since structural and organizational characteristics were largely similar between PICU 1 and PICU 2, the observed difference may be related to unit-specific factors such as local safety culture, adherence behaviors among permanent nursing staff, team dynamics, or differences in day-to-day CP reinforcement. Although consultant physicians were the same in both PICUs, resident physicians rotated monthly, which may also have contributed to variability in compliance practices between units. These findings suggest that CP adherence may be influenced not only by staffing and workload but also by behavioral and cultural factors within individual units.

No significant association was found between compliance and the type of patient care activity in the present study. This finding supports the idea that CP practices are performed as a routine behavior associated with room entry rather than being determined by the type of procedure performed. Previous observational studies have shown that only approximately two-thirds of HCWs entering CP rooms actually touched the patient or surrounding surfaces, while contact between clothing or gowns and the patient environment occurred in less than half of encounters.^12^ One possible explanation is that current CP recommendations generally promote the use of PPE for every room entry, regardless of the likelihood of actual physical contact or exposure. However, recommendations regarding PPE use in situations without direct patient contact differ between centers; some institutions do not recommend PPE use when entering the patient room without patient contact, whereas others do.^7^ It has been suggested that tailoring glove and gown use according to actual contact exposure, rather than universally requiring PPE for every room entry, could substantially reduce PPE consumption, time burden, cost, and environmental impact.^12^ The absence of higher compliance during invasive or high-contact procedures in this study may indicate that the requirement to wear PPE at every room entry reduces overall adherence and consequently leads to lower PPE use even during procedures involving closer or more frequent patient contact.

No significant association was found between compliance and microorganism type in this study. In the center where this study was conducted, CP were implemented for both VRE and MRSA. However, several centers have reported discontinuing CP for MRSA and VRE many years ago.^6^ Following the discontinuation of CP in patients with a history of MRSA colonization or infection, no long-term increase was observed in hospital-acquired MRSA incidence, MRSA-related device-associated infections, or surgical site infections.^26^ On the other hand, HCWs may perceive CP practices as burdensome, and these precautions may reduce the frequency of patient visits by HCWs.^6^ Nevertheless, carbapenem-resistant Gram-negative organisms, for which treatment options are limited, remain a major clinical concern, and the implementation of CP for these patients is critical to preventing transmission. Therefore, limiting CP practices, which are already perceived as burdensome by HCWs, to indications supported by scientific evidence may improve adherence in situations where such precautions are truly necessary. For example, reconsidering the routine use of CP for MRSA and VRE in the center where this study was conducted may contribute to better compliance with CP when genuinely indicated.

It has previously been reported that compliance with infection control precautions does not differ significantly between medical intensive care and surgical areas.^27^ Similarly, in the present study, no significant difference in compliance was found between PICU 1 and PSCU. However, in clinical practice, the workload and patient care dynamics of PSCU and PICU may differ considerably over time. In particular, PSCU may involve increased postoperative patient monitoring, a higher frequency of invasive procedures, and shorter durations of intensive care follow-up, whereas PICU care is often characterized by prolonged management of critically ill patients, complex medical treatments, and frequent HCW–patient contact. Despite these differences, the observation of similar compliance rates suggests that infection control practices may be influenced more by institutional habits, consistency of CP implementation, and unit-based adherence patterns than by the immediate clinical demands of the units.

This study has several limitations. First, observations were not concealed, which may have introduced a Hawthorne effect, whereby healthcare workers may modify their behavior because they are aware of being observed, potentially leading to an overestimation of compliance. However, observations were conducted during routine clinical care in an attempt to minimize behavioral modification. Second, repeated observations of the same HCWs were included, and disproportionate observation of individuals with lower compliance may have influenced the overall compliance rates. Third, the ordinal logistic regression model explained only a limited proportion of the variance in compliance levels (Nagelkerke *R* ^2^ = .072), suggesting that adherence to contact precautions is a multifactorial behavior influenced by additional unmeasured factors. Variables such as workload, local safety culture, team dynamics, staffing patterns, and perceived infection risk were not evaluated and may have contributed to variability in compliance. Despite these limitations, the prospective design and direct observation approach provided valuable real-world insights into adherence to contact precautions in pediatric intensive care settings.

In conclusion, adherence to CP in pediatric intensive care settings was suboptimal, with substantial variability across HCW groups and intensive care units. Given the limited evidence regarding CP practices in pediatric healthcare settings, our findings provide additional insight into the factors associated with adherence and identify opportunities for targeted institutional quality improvement. CP indications should be regularly updated in accordance with current literature and guidelines, while unnecessary use of CP should be avoided.

## Supporting information

10.1017/ash.2026.10902.sm001 Bayhan et al. supplementary material Bayhan et al. supplementary material

## Supplementary material

The supplementary material for this article can be found at https://doi.org/10.1017/ash.2026.10902.

## Table 1. Long description

A table with 11 rows and 5 columns comparing contact precaution compliance levels among healthcare workers. The columns are labeled Low compliance (n=57), Medium compliance (n=200), Full compliance (n=112), and P-value. The rows are labeled with different categories: Sex (Female, Male), Professional category (Nurse, Cleaner, Doctor, Intern doctor, Other personnel), Contact Time (12:30 and earlier, 12:31 and later), Microorganisms (Gram-negative, Gram-positive, Candidozyma auris, Viruses), ICU (PICU 1, PICU 2, PSCU), ICU Type (Pediatric, Surgical), and Types of patient contact activities (Minimal physical contact, Non-invasive physical contact, Invasive procedures or contact with body fluids, Aerosol-generating procedures). Each cell contains the number and percentage of observations for each category and compliance level. Notable trends include significant associations between compliance levels and professional category (P=0.022), ICU unit (P<0.001), and ICU type (P=0.014).

Navigate back to Table 1..

## Table 2. Long description

A table with six rows and five columns. The columns are labeled Variables, β, OR(95% CI), and p value. The rows are labeled Nurse vs other personnel, Cleaner vs other personnel, Doctor vs other personnel, Intern vs other personnel, PICU 1 vs PSCU, and PICU 2 vs PSCU. Row 1: Nurse vs other personnel, β 1.565, OR(95% CI) 4.78(1.20-19.08), p value .027. Row 2: Cleaner vs other personnel, β 1.126, OR(95% CI) 3.08(.70-13.61), p value .137. Row 3: Doctor vs other personnel, β .806, OR(95% CI) 2.24(.52-9.61), p value .278. Row 4: Intern vs other personnel, β 1.760, OR(95% CI) 5.81(1.14-29.60), p value .034. Row 5: PICU 1 vs PSCU, β .451, OR(95% CI) 1.57(.77-3.19), p value .212. Row 6: PICU 2 vs PSCU, β 1.003, OR(95% CI) 2.73(1.37-5.43), p value .004.

Navigate back to Table 2..

## Table 3. Long description

A table comparing compliance scores for different identified pathogens. The table has three columns: Identified pathogen, n, and Median (range). It contains eight rows of data. Row 1: Pseudomonas spp. (MDR/CR), 133, 4 (3-5). Row 2: VRE, 32, 3.5 (2-5). Row 3: Respiratory viruses, 103, 4 (3-5). Row 4: CRE, 13, 4 (3-5). Row 5: MRSA, 29, 4 (2-5). Row 6: Scarlet fever, 20, 4 (4-5). Row 7: Acinetobacter spp. (MDR/CR), 1, 1 (1-1). Row 8: Candidozyma auris, 7, 4 (3-5).

Navigate back to Table 3..
