# Supplementary material for: Evaluation of healthcare workers’ compliance with contact isolation precautions and associated factors in pediatric and pediatric surgery intensive care units
Source: Antimicrob Steward Healthc Epidemiol. 2026 Oct 15;6(1):e333. doi: 10.1017/ash.2026.10902 (PMC13760302; doi:10.1017/ash.2026.10902)
Supplement: Supplementary file 1 [file S2732494X26109024sup001.docx]

Supplementary Table 1. Details of patient care activities across four categories

Minimal Physical Contact
Physical Contact (Non-invasive)
Invasive / Body Fluid Exposure
Aerosol-Generating Procedures
Vital sign check
Repositioning patient
Administering medication
Aspiration
Check up
Changing bed sheets
Eye examination
COVID test
Ventilator settings changing
Administering formula milk
Establishing IV access
Respiratory failure intervention
ECG
Adjusting oxygen cannula
Changing diapers
Stabilizing ventilator tube
Prepare patient for Echochardiography
Applying bandage
Checking pus in drain area
Checking ventilator tube placement
Checking ventilator
Physical examination
Taking blood sample
Intubation
Assisting other staff

Blood transfusion
Inserting tube into patient
Entering room without touching
Wake up patient
IV medication
Setting up portable ventilator

Blood sugar measurement
Injection
Adjusting ventilator settings


Reconnecting patient to ventilator


Wound care
Repositioning breathing tube


Nasogastric tube yerleştirme
Checking tracheostomy tube


Draining tubes
Tracheostomy care


Adjusting ventilator


Assisting ventilator adjustment

Ultrasound

Setting up HFNC

Cleaning after ultrasound

Adjusting HFNC

Preparing patient for examination
Changing bandage
Performing CPR

Administering formula
Intravenous cannulation


Postural drainage
Central catheter insertion


Applying oxygen nasal cannula


Urinary catheter insertion


Hygiene care
Removing NG tube


Administration of blood products


Placing inhaler
Adjusting IV placement


Assisting bed bath
Bed bath


Assisting patient movement
Changing urinary catheter bag


Echochardiography
Readjusting NG tube


Checking PEG tube


Checking chest tube drainage


Applying topical medication


NG stabilization


Guiding X-ray while holding patient
Changing IV


Cleaning patient
Cleaning patient’s mouth


Wiping patient’s eyes
Checking IV access


Replacing IV bag
Checking urinary catheter


Wiping patient’s eyes


Checking IV placement
